# The Global Threat of New and Reemerging Infectious Diseases: Reconciling U.S. National Security and Public Health Policy

**DOI:** 10.3201/eid0909.030442

**Published:** 2003-09

**Authors:** Patrick J. McConnon

**Affiliations:** * Council of State and Territorial Epidemiologists, Atlanta, Georgia, USA

Brower and Chalk, authors of The Global Threat of New and Reemerging Infectious Diseases: Reconciling U.S. National Security and Public Health Policy, describe their book’s purpose as examining “the changing nature of security” and focusing on “the threat of infectious diseases.” There are many examples in today’s world where the intersection of threats to public health and national security should direct the attention of policymakers, security and public health strategists, and the systems that support each toward an organized response.

The authors use two case studies: HIV/AIDS in South Africa and the U.S. public health response system. The first case, in South Africa, illustrates how a single microbial agent can undermine the economic, social, and medical underpinnings of a developed country. The second study shows the negative effect of newly emerging diseases such as HIV/AIDS, Hantavirus infection, West Nile virus infection, Creutzfeldt-Jakob disease, and intentionally released agents (*Bacillus anthracis*). This study demonstrates how events can overload the public health response system and weaken public confidence in its government. The reader can easily conclude that the intersection of disease and national security can be dangerously destabilizing and seriously undermine a nation’s social, economic, and political order. The current outbreak of severe acute respiratory syndrome reinterates the global nature and warp speed of emerging infections.

In their summary and conclusions, the authors provide recommendations for policymakers addressing both public health and security issues. The thrust of the authors’ conclusions is to push policymakers and strategists to actions that strengthen the infrastructure of a public health response system and broaden the traditional definition of national security to include the impact of naturally occurring and intentionally released microbial agents.

The authors present a compelling case study for HIV/AIDS in South Africa, where an emerging disease has gone unchecked and is having a devastating effect on a developed country. The case study of the U.S. public health response system is interesting and thoughtfully presented but lacks sufficient and carefully documented detail to aid the reader in drawing conclusions and formulating solutions. Unsubstantiated or incorrect examples also detract from the overall presentation of this case study. For instance, the contention that lack of good communications with area physicians and hospitals resulted in the deaths of postal workers in the fall 2001 anthrax crisis is not supported by the author’s reference or by any other authoritative materials known to this reviewer. In the public health response case study, the authors provide broad recommendations aimed at strengthening the public health infrastructure. Also included is an excellent summary of the current status of efforts begun in the mid-1990s in the United States to address the infrastructure of public health. The recommendations are presented in such a way that the shortcomings of the system can be addressed in critical areas, including a well-trained public health workforce; interagency coordination; private sector, hospital, and emergency response integration in public health; technical and educational interventions; and domestic and global investment in public health.

Brower and Chalk’s book is a powerful and useful argument for the urgent need to integrate and streamline public health and national security strategies.

